# Sepsis Due to Achromobacter xylosoxidans in a Tertiary Care Centre: Case Series

**DOI:** 10.7759/cureus.42052

**Published:** 2023-07-17

**Authors:** Gargi Mudey, Radha Kunjalwar, Gaurav Sahu, Shital M Mahajan, Supriya Meshram

**Affiliations:** 1 Department of Microbiology, Jawaharlal Nehru Medical College, Datta Meghe Institute of Higher Education and Research, Wardha, IND; 2 Department of Medicine, Jawaharlal Nehru Medical College, Datta Meghe Institute of Higher Education and Research, Wardha, IND

**Keywords:** immunocompromised patient, multi-drug resistant, gram-negative organism, bacteremia, achromobacter xylosoxidans

## Abstract

*Achromobacter xylosoxidans*, also known as *Alcaligenes xylosoxidans*, is a low-virulence, non-fermenter gram-negative bacillus mainly found in marine environments. We report a detailed series of four high-risk cases of septicemia with the common variable of positive blood cultures for *A. xylosoxidans*. All four blood isolates were multi-drug resistant and susceptible to meropenem and trimethoprim-sulfamethoxazole. Two patients responded well to the treatment with meropenem and trimethoprim-sulfamethoxazole and two patients died. It should never be assumed that *Achromobacter* is a contaminant even though it is relatively infrequently isolated from clinical samples. This infection can progress to fatal bacteremia, even in otherwise healthy people, and it can potentially cause severe conditions in premature infants. With only a limited number of antibiotics demonstrating bactericidal properties, the possibility of failure in empirical treatment is significant. As a result, it is important to have a precise comprehension of this uncommon yet deadly illness in order to increase the probability of successful treatment.

## Introduction

*Achromobacter xylosoxidans* is a non-fermenting gram-negative bacillus. Being closely associated with *Alcaligenes* species, it was also called *Alcaligenes xylosoxidans* [1]. Yabuuchi and Ohyama identified it in purulent ear discharge from chronic otitis media patients in 1971 [2].

It is a rare, low-virulence opportunistic pathogen. However, invasive infections such as bacteremia, meningitis, pulmonary abscess, peritonitis, urinary tract infections, prosthetic valve endocarditis, chronic otitis media, osteomyelitis, endophthalmitis, and septic arthritis have been documented in patients having cancer, immunoglobulin M (IgM) immunodeficiency, post-valve replacement patients, and newborns [3].

This bacterium often resides in aquatic sources in the environment, hospitals, and human guts, but it can also cause nosocomial infections and diseases acquired in the community. This bacterium causes invasive infections that are highly lethal in immunocompromised people and newborns [4]. This report highlights four high-risk patients with blood cultures positive for *A. xylosoxidans* by BacT/ALERT 3D (bioMérieux, Durham, NC, USA) and VITEK-2 (bioMérieux, Marcy-l'Étoile, France) automated systems.

## Case presentation

We discuss four cases that were reported to the Acharya Vinoba Bhave Rural Hospital (AVBRH), a tertiary care rural hospital situated in central India majorly catering to the population of the Vidarbha region of Maharashtra, India.

Case 1

A 30-year-old male, with a known case of sickle cell disease with SS pattern, was admitted to the medicine ward with complaints of chest pain and abdominal discomfort for 10 days. He reported no recent contact with sick individuals or travel history. On examination, the patient was febrile to touch (39.2°C/102.6°F), developed tachycardia, and in mild respiratory distress. Physical examination revealed scleral icterus, moderate splenomegaly, and diffuse abdominal tenderness.

On the day of admission, lab investigations revealed a haemoglobin level of 8.8 gm/dl, white blood cell count (WBCs) of 7100/mm3, and platelets of 96,000/cumm. On day 3, haemoglobin level dropped to 3.1 gm/dl, WBCs to 11,900/mm3, and platelets 24,000/cumm. Given severe anaemia and thrombocytopenia, seven units of packed red blood cells (PBRC) and four random donor platelets were transfused. Blood smear examination demonstrated microcytic hypochromic red blood cells (RBCs) with anisopoikilocytosis showing few microcytes, pencil cells, and nucleated red blood cells (RBCs). The peripheral blood was sent for culture, and empirical broad-spectrum antibiotic Cifran CT was initiated.

**Figure 1:**
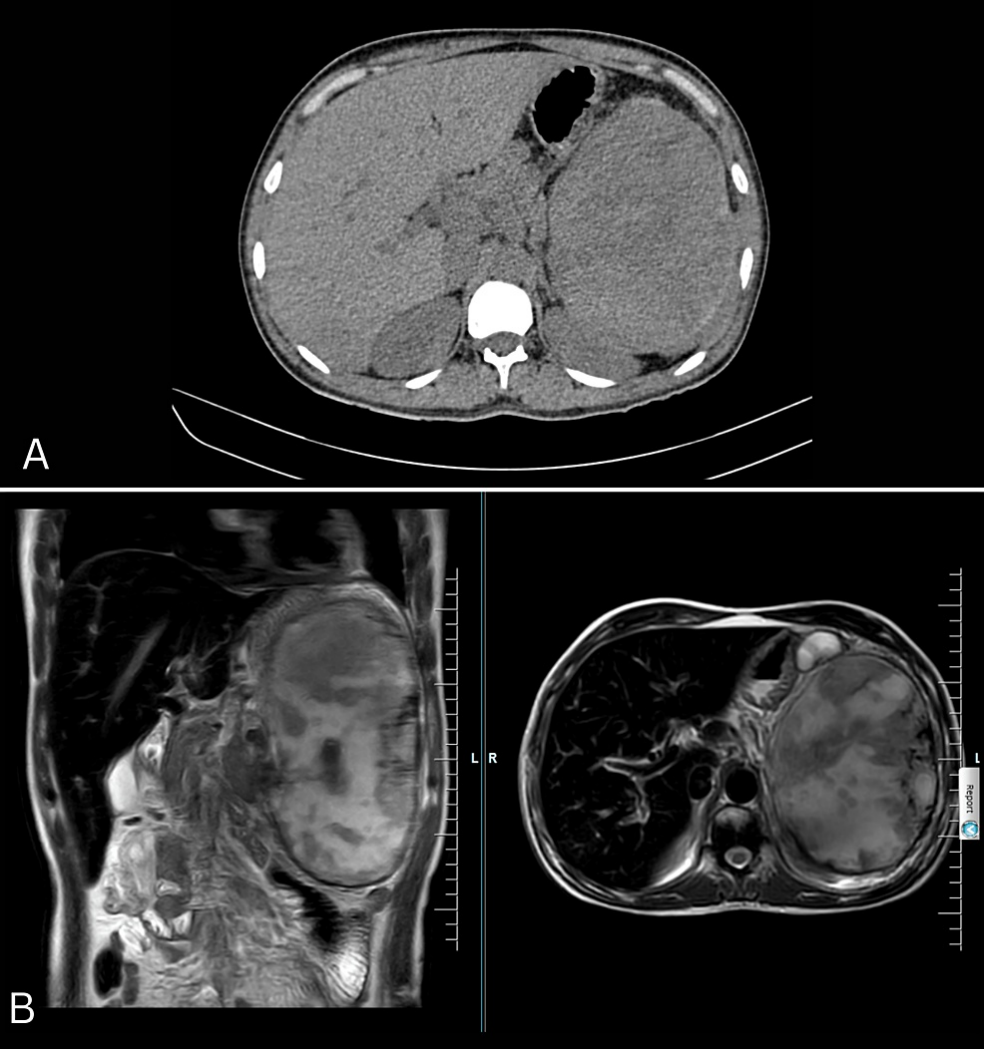
A) CECT abdomen showing hemangioma in the spleen with splenomegaly and hepatomegaly; B) MRCP suggests cholelithiasis, hepatomegaly with focal haemorrhages, splenomegaly with splenic infarcts with haemorrhage CECT - Contrast-enhanced computed tomography, MRCP - Magnetic resonance cholangiopancreatography

Chest X-ray and electrocardiogram (ECG) revealed no obvious abnormality. Ultrasonogram (USG) (abdomen pelvis) was suggestive of massive splenomegaly, abnormal aneurysmal dilatation in spleen parenchyma, and splenic infarcts. Contrast-enhanced computed tomography (CECT) abdomen was suggestive of a large hemangioma in the spleen for which splenic artery embolisation was done after the interventional radiologist's opinion. Magnetic resonance cholangiopancreatography (MRCP) was done after surgery opinion, suggesting cholelithiasis, hepatomegaly with focal haemorrhages, splenomegaly with splenic infarcts with haemorrhage, for which splenectomy was scheduled (Figure 1A, 1B).

On day 13, the patient's condition deteriorated rapidly. He developed worsening tachycardia, hypotension, and acute respiratory distress syndrome (ARDS). Repeat laboratory tests showed worsening anaemia, progressive leukocytosis (WBC count of 17,400/mm3), and evidence of disseminated intravascular coagulation (DIC).

**Figure 2:**
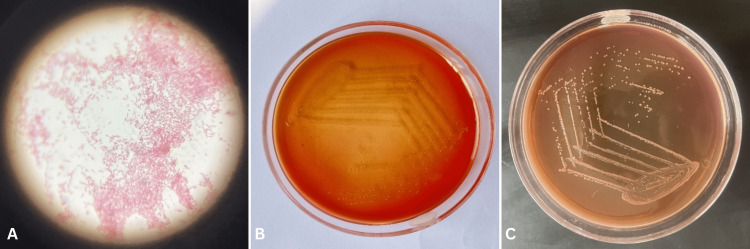
A) Gram stain of culture smear showing gram-negative bacilli; B) Blood agar showing colonies of Acetobacter xylosoxidans; C) Non-lactose fermenting colonies of A. xylosoxidans on MacConkey agar

On day 14, Blood culture was positive for *A. xylosoxidans* (Figure 2A, 2C). Intravenous fluids, vasopressors, and respiratory support were initiated promptly. The patient was transferred to the intensive care unit for further management. The next day, as scheduled, a splenectomy was done. Meropenem and piperacillin-tazobactam injections were administered to the patient. After receiving the proper antimicrobial medication, the clinical picture and haematological parameters both significantly improved.

Case 2

A 35-year-old female patient was brought to the hospital with chief complaints of breathlessness for 15 days, aggravated since 2 days, cough since 15 days, on and off fever since 1 month, swelling and erythema over bilateral lower limb since 45 days which turned into fluid-filled blisters and oozing ulcers since 3 days. No history of cold, loose stools, pain in the abdomen, hematemesis, loss of consciousness, seizure, chest pain, palpitation, or vertigo. No history of diabetes mellitus, systemic hypertension, bronchial asthma, or tuberculosis. The patient was a known case of mitral valve replacement 3 years back on tab Acitrom 1 mg - 0.5 mg alternate days.

On general examination, the patient's general condition was poor, afebrile, pulse was 108 per minute, blood pressure was 110/70 mm of hg, and oxygen saturation (SpO2) at 98% on BiPAP support.

**Figure 3:**
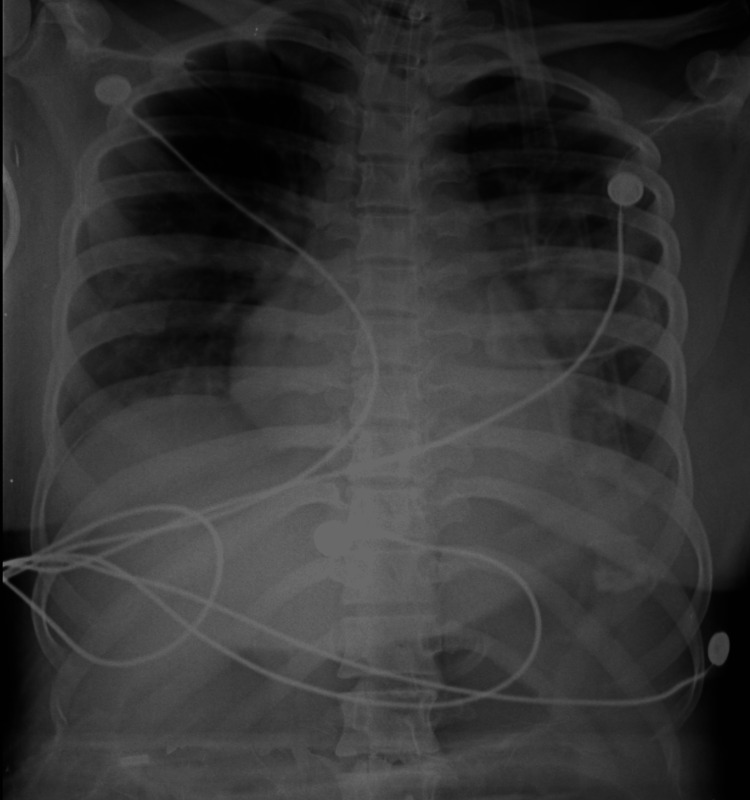
Chest X-ray showing bilateral lower lobe infiltrates

On systemic examination, cardiovascular system: first heart sound (S1) was metallic click and variable; respiratory system: bilateral air entry equal; per abdomen: soft non-tender, central nervous system: conscious oriented. Laboratory investigations revealed haemoglobin levels to be 6.8 gm/dl and leukocytosis (WBC count of 17,000/mm3) with neutrophilic predominance. Arterial blood gas analysis showed respiratory alkalosis. Chest X-ray demonstrated bilateral lower lobe infiltrates. Empirical broad-spectrum antibiotics, including ceftriaxone and azithromycin injections, were initiated, and the patient was admitted to the intensive care unit for further management (Figure 3).

Over the next 24 hours, the patient's condition deteriorated rapidly. She developed hypotension, worsening respiratory distress, and signs of septic shock. Blood cultures were obtained, and vasopressor support was initiated. After 48 hours, the blood culture was positive for *A. xylosoxidans*. The antibiotic regimen was adjusted, and intravenous meropenem injection was initiated for 10 days based on susceptibility testing. Oral trimethoprim-sulfamethoxazole was prescribed on discharge post-recovery. After 21 days of therapy, a follow-up revealed a marked improvement in the patient's condition.

Case 3

A 1-day-old premature male neonate weighing 1.2 kg was delivered by emergency lower segment cesarean section (LSCS) due to fetal distress to a primigravida mother at 29 weeks having twin pregnancy. Prenatal ultrasound revealed total anomalous pulmonary venous connection (TAPVC). The neonate was admitted to the neonatal intensive care unit (NICU) for close monitoring and management. The baby was kept on nasal prongs and still was unable to maintain saturation hence was intubated with ET No. 3. Laboratory investigations on admission revealed haemoglobin levels to be 11 gm/dl, WBC 11,300/mm3, platelets 1.2/cumm, and calcium 7.5 mg/dl. Amikacin and cefotaxime injections were started.

On the second day, 2D-ECHO confirmed TAPVC, isolated pulmonary venous stenosis on the left side with grossly dilated right atrium and right ventricle, and severe pulmonary hypertension. Prostaglandin infusion was initiated to maintain ductal patency, and nitric oxide therapy was started for pulmonary vasodilation. The neonate developed clinical signs of sepsis, including raised temperature (>39°C), tachycardia, and poor perfusion. Blood cultures were obtained, and vancomycin, fluconazole, and midazolam injections were added. Despite aggressive management, the neonate's condition deteriorated rapidly.

On the third day, the baby was transfused 1 unit of packed Red Blood Cells as the haemoglobin levels dropped to 6.4 gm/dl. Blood culture results were positive for *A. xylosoxidans*, indicating a severe opportunistic infection. The antibiotic regimen was adjusted based on susceptibility testing, and the neonate received intravenous meropenem. However, despite all measures, the neonate's clinical status continued to worsen. He developed refractory hypotension, disseminated intravascular coagulation (DIC), and multiorgan dysfunction.

Up to the fourth day, the baby could maintain saturation of the ventilator settings without any episode suggestive of respiratory distress; however, one episode of desaturation was recorded, and adrenaline injection of 0.5 mg was given. On the fifth day, saturation was progressively deteriorating, along with episodes of bradycardia. Later vitals were recorded as heart rate-30, pulse pressure was not felt well, and SpO2 - 0. Cardiopulmonary resuscitative (CPR) measures with bag and mask ventilation and adrenaline bolus were continued. Despite all the resuscitation efforts, the baby’s condition progressively worsened, and could not be revived and was declared dead.

Case 4

A 15-year-old male with a history of double outlet right ventricle (DORV) and a large ventricular septal defect (VSD) diagnosed 8 months ago was admitted to the pediatric intensive care unit (PICU) with complaints of fever, breathlessness, and pain in his upper and lower limbs. On examination, the patient appeared pale, with tachycardia, tachypnea, and signs of congestive cardiac failure, including hepatomegaly and peripheral oedema. The patient was on Lasix since one and a half months of age, Pentid started at 3 years of age, and Dixin started at 1.5 months of age

Cardiovascular examination revealed normal first heart sound (S1), loud second heart sound (S2), and loud pulmonary valve closure (P2) with grade 3/6 holosystolic murmur along the left sternal border. Abdominal examination was normal, and higher function tests showed altered sensorium. The rest of the systemic examination was unremarkable.

On the second day, laboratory investigations were done, which revealed haemoglobin levels to be 20.9 gm/dL, WBCs to be 5600/mm3, and platelets, 2.1 /cumm. The patient had an episode of cyanotic spell with generalised tonic-clonic seizure (GTCS) for which metoprolol and sodium tricarbonate injections were given stat along with the exchange of 50 ml blood and phenytoin injection. Calcium gluconate and magnesium sulphate injections were given for hypocalcemia and hypomagnesemia. CT head and brain scan were done, suggesting no obvious abnormality in brain parenchyma.

**Figure 4:**
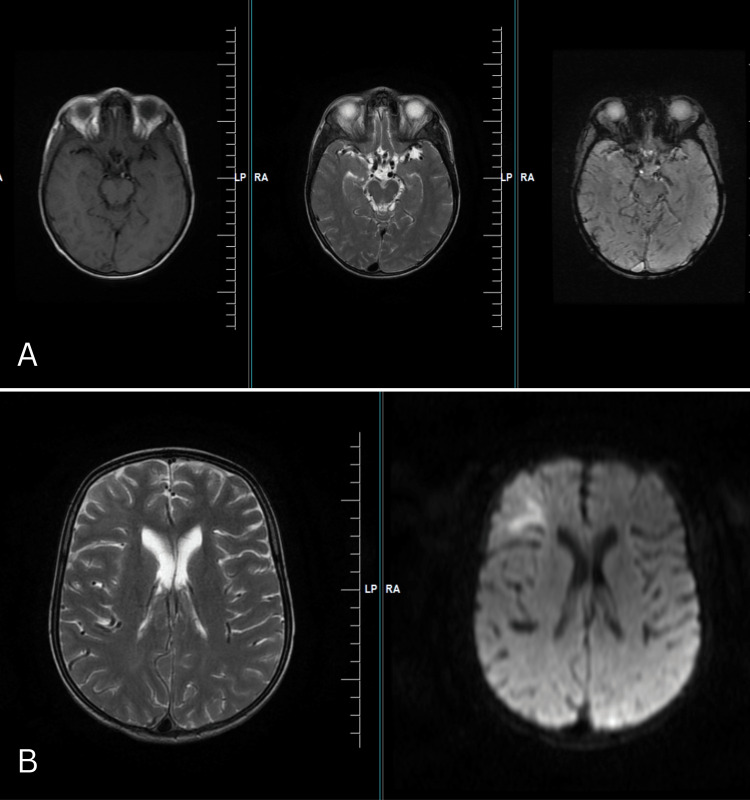
A) Focal subcortical hyperintensity in T2-weighted MRI in right frontal region showing restricted diffusion; B) Prominent dilated intracranial vessels with flow void on T1-weighted MRI and T2-weighted MRI, along with punctate blooming on GRE MRI - Magnetic resonance imaging GRE - Gradient recall echo

Arterial blood gas analysis showed hypoxemia and respiratory alkalosis. Chest X-ray revealed cardiomegaly, pulmonary plethora, and decreased lung perfusion on the left side. CT head and brain scan were done, suggesting no obvious abnormality in brain parenchyma. Given the patient's clinical presentation and history of congenital heart disease, blood cultures were obtained. Empirical broad-spectrum antibiotic therapy with intravenous ceftriaxone injection was initiated. The patient was also started on oxygen supplementation and diuretics to manage respiratory distress and fluid overload. A magnetic resonance imaging (MRI) brain was done, which showed prominence of intracranial vasculature suggestive of polycythemia and postictal changes in the right frontal region (Figure 4).

**Figure 5:**
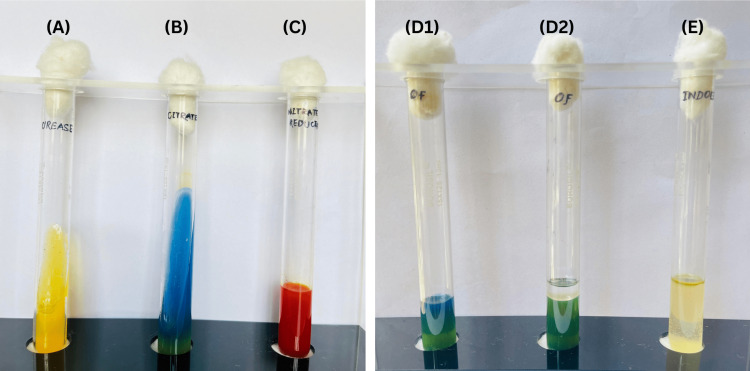
Biochemical reactions of A. xylosoxidans (A - Urease negative, B - Citrate positive, C - Nitrate reduction positive, D1,2 - OF negative, E - Indole negative) OF - Oxidative-fermentation test

On day 5, the blood culture report was positive for *A. xylosoxidans*. Sensitivity testing showed susceptibility to meropenem and amikacin injections and the antibiotic regimen was modified accordingly. Biochemical reactions were also tested (Figure 5). A transthoracic echocardiogram revealed no evidence of endocarditis or abscess formation.

Despite appropriate antibiotic therapy, the patient's clinical condition deteriorated, Sensorium was depressed and respiratory distress was present. Intravenous fluid D10, D25, and NS, injection noradrenaline, nebulisation with salbutamol were started. The patient had evident respiratory distress and did not maintain saturation and was intubated. The patient was kept nil by mouth (NBM), intravenous fluid DNS with KCl started, and a complete blood count (CBC) was done. Haemoglobin was 19.6 gm/dL, WBCs were 10,100/cumm, platelets were 27,000/cumm, hematocrit (HCT) 61.1, Na - 137 mmol/L, K - 4.3 mEq/L, Mg - 4.6 mg/dl, P - 3.7 mg/dl, Ca - 7.8 mg/dl. The patient’s sensorium was poor and was cyanosed with cold extremities. The patient went into bradycardia with a heart rate of 46 bpm and SpO2 of 40%. Bag and mask ventilation was started along with cardiopulmonary resuscitation (CPR). Adrenaline 2.4 mL was administered and CPR was continued. The patient was declared dead after all attempts at resuscitation failed.

*A. xylosoxidans* isolates in all four cases were subjected to a wide array of antibiotics to check their susceptibility which is summarised in Table 1.

**Table 1:** Minimum inhibitory concentration of antibiotics tested MIC - Minimum inhibitory concentration, S - Sensitive, I - Intermediate, R - Resistant

	Antibiotics												
Cases	PIPERACILLIN + TAZOBACTAM	CEFTAZIDIME	CEFOPERAZONE/SULBACTAM	CEFEPIME	AZTREONAM	IMIPENEM	MEROPENEM	AMIKACIN	GENTAMYCIN	CIPROFLOXACIN	LEVOFLOXACIN	MINOCYCLINE	TRIMETHOPRIM/SULFAMETHOXAZOLE
Case 1- MIC- (µg/ml)	4 [S]	8 [S]	32 [I]	16 [I]	≥64 [R]	2 [S]	≤0.25 [S]	≥64 [R]	≥16 [R]	≥4 [R]	4 [I]	8 [I]	≤20 [S]
Case 2- MIC- (µg/ml)	≥128 [R]	≥64 [R]	≥64 [R]	16 [I]	≥64 [R]	8 [I]	1 [S]	≥64 [R]	≥16 [R]	2 [I]	4 [I]	8 [I]	40 [S]
Case 3- MIC- (µg/ml)	≥128 [R]	2 [S]	32 [I]	≥32 [R]	≥64 [R]	≥16 [R]	4 [S]	≥64 [R]	≥16 [R]	≥4 [R]	4 [I]	8 [I]	≤20 [S]
Case 4- MIC- (µg/ml)	≥128 [R]	≥64 [R]	≥64 [R]	≥64 [R]	≥64 [R]	1 [S]	0.5 [S]	≥64 [R]	≥16 [R]	≥4 [R]	4 [I]	8 [I]	≤20 [S]

All the radiological investigations (USG, CECT, MRCP, 2D-ECHO) in these cases were done to diagnose the presented case; however, specific emphasis was on analysing the rare bacterial isolates, that is *A. xylosoxidans* which were obtained from the blood cultures in all the four cases (Appendix A-D).

## Discussion

*A. xylosoxidans* is a gram-negative, flagellated, motile, aerobic, non-glucose fermenting bacillus. The clinically significant species are *xylosoxidans* and *denitrificans* [5]. It is commonly confused with other non-fermenters, which can affect the clinical management of patients substantially as most of the time they are considered environmental contaminants [1].

 The first pancreatic pseudocyst report and local wound infection of metastatic ductal carcinoma caused to *A. xylosoxidans* have been reported by Eshwara et al. [1].

Turel O. et al. reported bacteremia due to *A. xylosoxidans* in 22 neonates in Istanbul, Turkey (2013), showed resistance to amikacin and gentamycin (100%), sensitivity to meropenem and trimethoprim-sulfamethoxazole (100%), piperacillin-tazobactam (91%), ceftazidime and ciprofloxacin (82%), is intermediate to Cefepime (9%). Meropenem alone (9.7%), meropenem in combination with other antibiotics (ciprofloxacin, ceftazidime, and piperacillin-tazobactam in 50%, 22.7%, and 13.6% of patients, respectively), or ciprofloxacin with ceftazidime (4.5%) were given to patients for a 10-21 days course. Three patients expired; the rest recovered [6].

Another study by Otta S. et al. in Bhubaneswar, India (2014) reported *Achromobacter* in an acute pancreatitis patient and was found resistant to third- and fourth-generation cephalosporins, sensitive to piperacillin-tazobactam, meropenem and trimethoprim‑sulfamethoxazole and intermediate to imipenem, levofloxacin, and tigecycline. The patient improved when treated with amikacin and piperacillin-tazobactam [7].

Ucciferri C. et al. reported a case of a 47-year-old lady in Italy (2021) diagnosed with common variable immunodeficiency (CVID) with positive blood culture for *A. xylosoxidans* along with positive cultures from peripheral and Groshong central venous catheter (CVC) which had acquired resistance to piperacillin-tazobactam during the course of treatment and was later treated with meropenem 3 gm/day [8].

Barakat M. et al. in Doha, QAT (2022) pointed out the resistance to amikacin, cefepime, ciprofloxacin, levofloxacin, and gentamycin; sensitivity to piperacillin-tazobactam, meropenem, and trimethoprim-sulfamethoxazole; and intermediate pattern to ceftazidime in a patient with septicemia complicated by septic shock and multi-organ failure which led to death [9].

In our four cases, we found all the isolates were resistant to aztreonam, gentamycin, and amikacin; sensitive to meropenem and trimethoprim-sulfamethoxazole; and intermediate to levofloxacin and minocycline. Two patients recovered after treatment with meropenem and trimethoprim-sulfamethoxazole in our case. Quinolones exhibit poor activity against *Achromobacter spp.*, according to studies by Gomez-Cerezo J. et al. and Almuzara M. et al. [10,11]. In contrast, our four isolates were intermediate to levofloxacin with a minimum inhibitory concentration (MIC) of 4 µg ml-1.

Our report detailed a series of four high-risk cases of septicemia with the common variable of positive blood cultures for *A. xylosoxidans*. All the isolates were multi-drug resistant and sensitive to meropenem and trimethoprim-sulfamethoxazole. The sensitivity pattern is shown in Table 1. Rapid diagnosis and prompt treatment saved the lives of both patients. 

## Conclusions

Our findings contribute to a better understanding of the range of illnesses caused by the opportunistic pathogen *Achromobacter xylosoxidans*, which is a rare but significant infection source. Automation was crucial in the quick and accurate identification of such rare pathogens, which can be mistaken as contaminants, and in most cases, the prompt beginning of the proper antibiotic medication resulted in a favourable outcome. Both patients' lives were saved by quick diagnosis and prompt treatment. More research is required to evaluate the significance of various sources of infection in hospital units.

## Figures and Tables

**Figure 6:**
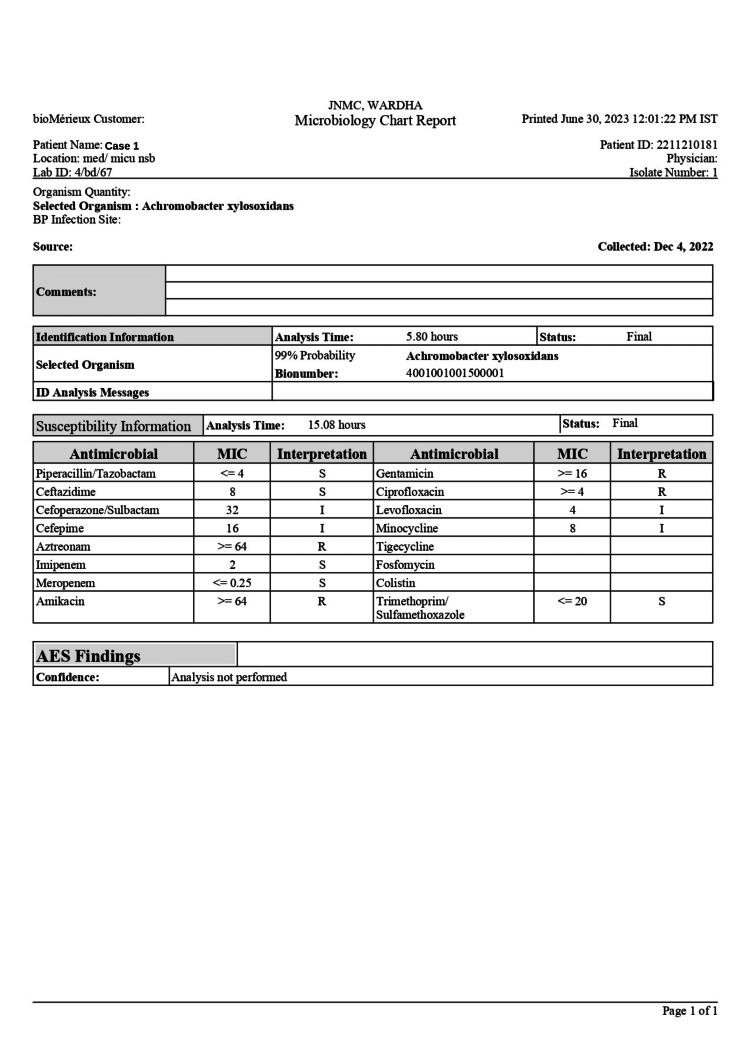
Identification and antimicrobial susceptibility test of multidrug-resistant Achromobacter xylosoxidans by VITEK-2 compact system for case 1 MIC - Minimum inhibitory concentration, AES - Advance Expert System

**Figure 7:**
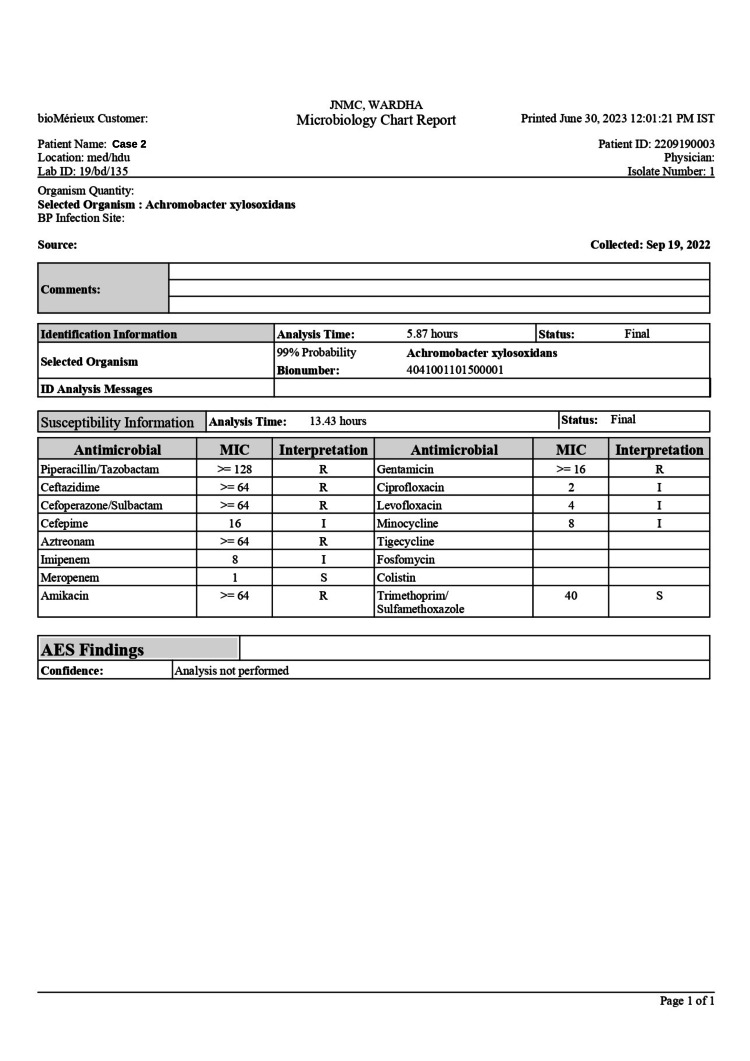
Identification and antimicrobial susceptibility test of multidrug-resistant Achromobacter xylosoxidans by VITEK-2 compact system for case 2 MIC - Minimum inhibitory concentration, AES - Advance Expert System

**Figure 8:**
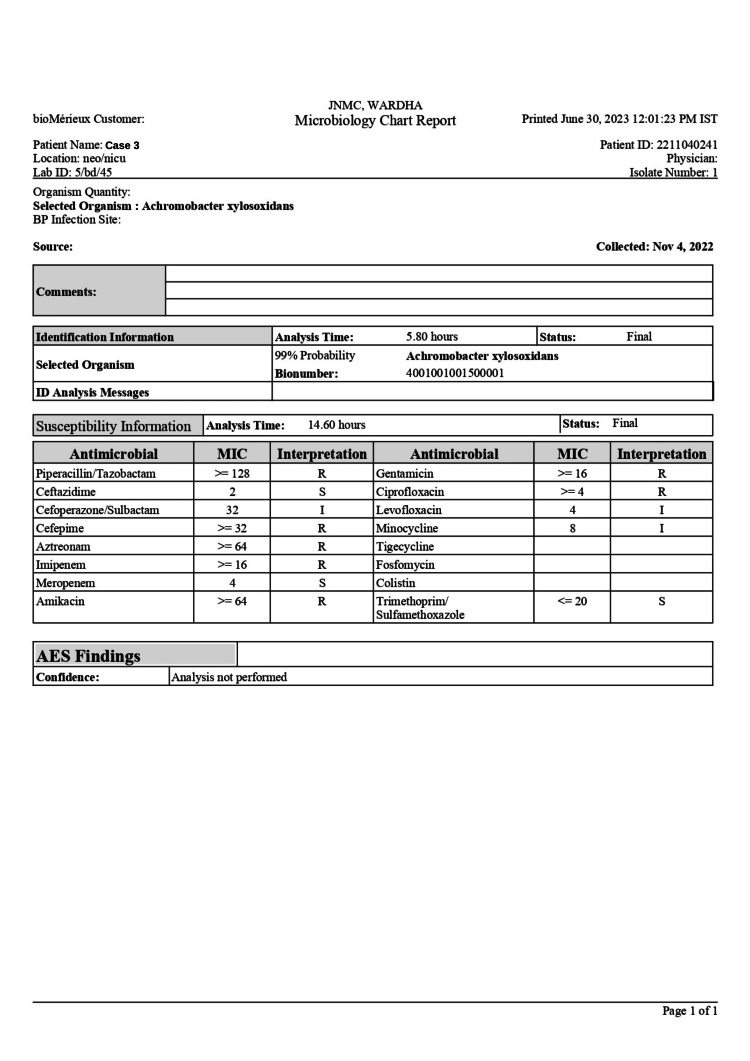
Identification and antimicrobial susceptibility test of multidrug-resistant Achromobacter xylosoxidans by VITEK-2 compact system for case 3 MIC - Minimum inhibitory concentration, AES - Advance Expert System

**Figure 9:**
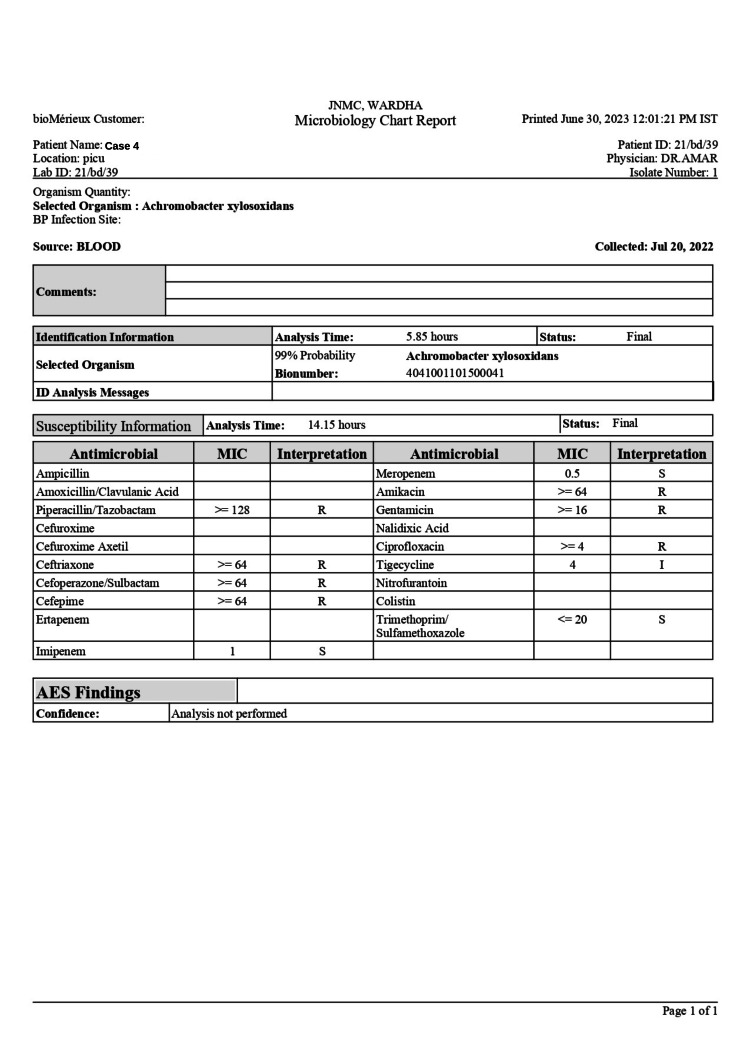
Identification and antimicrobial susceptibility test of multidrug-resistant Achromobacter xylosoxidans by VITEK-2 compact system for case 4 MIC - Minimum inhibitory concentration, AES - Advance Expert System

## Appendices

Appendix A 

Appendix B 

Appendix C 

Appendix D 
